# Editorial: The developing kidney: Perinatal aspects and relevance throughout life

**DOI:** 10.3389/fped.2022.990854

**Published:** 2022-07-28

**Authors:** Karel Allegaert, Silvia Iacobelli

**Affiliations:** ^1^Department of Development and Regeneration, KU Leuven, Leuven, Belgium; ^2^Department of Pharmaceutical and Pharmacological Sciences, KU Leuven, Leuven, Belgium; ^3^Child and Youth Institute, KU Leuven, Leuven, Belgium; ^4^Department of Clinical Pharmacy, Erasmus Medical Center (MC), Rotterdam, Netherlands; ^5^Réanimation Néonatale et Pédiatrique, Néonatologie, CHU La Réunion, Site Sud, Saint Pierre, France; ^6^Centre d'Études Périnatales de l'Océan Indien UR 7388, Université de la Réunion, Saint Pierre, France

**Keywords:** acute kidney injury, newborn, fetal, nephron number, perinatology

Human perinatal nephrology is a very diverse field in medicine, shared—among others—between obstetricians, neonatologists and nephrologists. Extremely low birth weight infants, babies with growth restriction, and specific subgroups (like asphyxia, cardiac bypass) of term neonates in the neonatal intensive care units (NICUs) are predisposed to acute kidney injury (AKI) and are prone to develop progressive renal failure at early age. Cases with congenital malformations of the kidney and urinary tract (CAKUT) are another specific group.

A holistic, multidisciplinary approach is needed to assess the degree and impact of kidney impairment in these vulnerable infants. This is because there is a wealth on observations that fetal and neonatal renal development is one of the main drivers of short and long-term outcome (Developmental Origins of Health and Disease, DOHaD). Furthermore, the variability in renal development and function should make us explore the impact of preventive or curative interventions, with disrupted nephrogenesis as key mechanism.

Nephrogenesis is the highly complex process that leads to the formation of nephrons as functioning units of the kidney, as illustrated in Figure 1, to result in maturation of glomerular filtration in fetal, neonatal life and beyond (1). The overarching paradigm relevant to this Research Topic is “the number of nephrons”, or how to prevent or avoid nephron deficit. It is hereby relevant to realize that formation of nephrons ceases at ~36 weeks of gestation. In case of preterm birth, whatever the gestational age, nephrogenesis ceases by the end of the first 40 days of life. Antenatal exposure to medicines, malnutrition, or other environmental factors, may result in congenital malformations or preterm birth, and may influence kidney development (2, 3).

**Figure 1:**
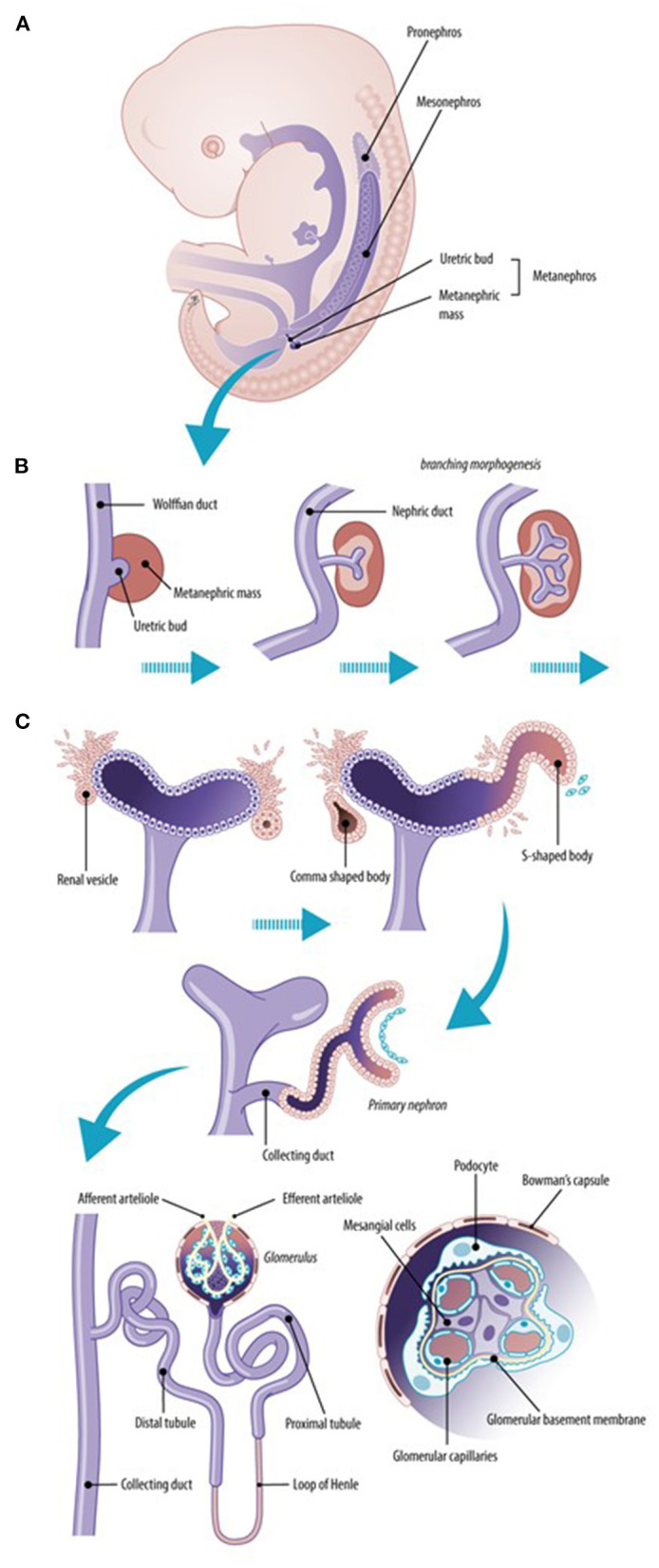
Normal renal development. The early ureteric bud interacts with the metanephric mesenchyme to form the pronephric duct (pronephros). The more caudal mesonephric ducts are the first “glomeruli–like” structures (mesonephros) that form transiently active filtration units while the metanephros is created **(A)**. Early development of the kidney implies branching morphogenesis **(B)** through the interaction between the ureteric bud and the metanephric mass. The metanephric mass undergoes mesenchymal–to–epithelial transformation to form nephrons, including the glomeruli and tubuli **(C)**. The final nephron number is attained at about 34–36 weeks of gestational age (10-fold variability, 200,000–2,000,000).

**Fetal kidney function** only marginally contributes to the overall fetal elimination clearance, as the placenta somewhat acts as “physiological dialysis construct”. Fetal urine, urinary flow (amniotic fluid) and its composition (including creatinine) reflect an increase in renal elimination capacity over gestational age, with 10-fold difference throughout fetal life. Maternal medicines exposure, maternal diseases (either or not pregnancy related), or malnutrition may impact the nephron number and/or kidney function (2). There are two papers in this Research Topic that focus on this aspect. First, Ezuruike et al. quantified fetal renal function, based on fetal urine production rate, fetal and amniotic fluid creatinine levels throughout pregnancy. The derived fetal urinary production rate and creatinine functions can be applied to assess fetal renal maturation, to predict fetal renal clearance and to explore the impact of mediators of these functions (Ezuruike et al.). One of these mediators is inflammatory syndromes, like chorioamnionitis. Along these lines, Hoogenboom et al. reported on kidney inflammation, podocyte damage and the pro-fibrotic changes in a fetal lamb chorioamnionitis (preterm intra-amniotic lipopolysaccharide injection) model. This animal model provides mechanistic information on the link between antenatal inflammation and the subsequent preterm-associated kidney disease (Hoogenboom et al.).

Related to **neonatal kidney function**, relevant progress to capture the (patho)physiology of renal impairment and AKI has been made. Jetton and Askenazi (4) constructed an AKI definition (neonatal modified KDIGO) specific for use in neonates. Besides urine output indicators, this definition is based on a creatinine increase ≥ 0.3 mg/dl or 1.5–1.9-fold increase from “baseline” within 48 h or 7 days, respectively (*stage 1*), a creatinine increase from “baseline ≥ 2–2.9” (*stage 2*), or creatinine > 2.5 mg/dl, renal ≥ 3-fold from “baseline” (*stage 3*) (5). It is hereby suggested that a serum creatinine of 2.5 mg/dl reflects an estimated glomerular filtration rate <10 ml/min/1.73 m^2^. However, such “static” definition do not sufficiently well cover the dynamics in creatinine patterns. Consequently, there are still limitations as this AKI definition does not fully discriminate between physiology and pathophysiology of renal function and creatinine trends (6). This resulted in better identification of risk groups (“precision medicine”), development of prediction models (like urinary neutrophil gelatinase-associated lipocalin (NGAL) after cardiac surgery) (7). Alternatively, a suggestion to consider a centile approach (somewhat similar to commonly used growth charts) over postnatal age has been applied to extreme low birth weight (ELBW) infants, and more recently, to neonates treated with therapeutic hypothermia for neonatal encephalopathy (6, 8).

Likewise, the baby NINJA (Nephrotoxic Injury Negated by Just-in-Time Action) study illustrated the feasibility of (secondary) prevention, as this study documented that a reduction of exposure of nephrotoxic medicines (duration of treatment) resulted in a decrease in AKI incidence and severity (9). As discussed by DeFreitas et al., oxidative stress has a negative effect on the developing kidney, as it relates to development of hypertension and kidney injury in animal model. In contrast, clinical research addressing the implications of oxidative stress in the developing kidney is less developed than that of neurodevelopmental and respiratory conditions of preterm infants (DeFreitas et al.). These authors therefore call for additional efforts to study the relevance of this mechanism on renal outcome, in addition to the more commonly studies neurodevelopmental and respiratory outcome (DeFreitas et al.).

**Long term renal outcome** related to these pre- or postnatal exposures are therefore crucial to subsequently link associated covariates to secondary prevention strategies (2, 3). To illustrate feasibility, a case-control study in former ELBW infants quantified a shift of about one standard deviation to poorer eGFR in former ELBW cases in late childhood. Unfortunately, we failed to link the presence of neonatal AKI, neonatal creatinine trends, or expected risk factors (ibuprofen, pre- or postnatal steroids) to poorer eGFR, so that implementation of targeted secondary prevention or follow-up programs remains difficult (10). Along these lines, the paper of Rypdal et al. documented the absence of an association between the extent of neonatal gentamicin exposure and subsequent signs of subclinical nephrotoxicity (urinary protein-creatine ratio, albumin-creatinine ratio, kidney injury molecule-1, N-acetyl-beta-D-glucosaminidase normalized form urine creatinine) or blood pressure at school age in 222 cases. In addition to the compound specific findings, the non-invasive methodological approach may also be helpful to other researchers, considering other perinatal covariates of relevance (Rypdal et al.).

Overall the different papers in this Research Topic added ideas and provided pieces of new information. We are confident that the illustrations provided in this Research Topic will further boost the clinical research on the perinatal aspects and relevance of the developing kidney throughout life and holds the promise to further improve neonatal management, quality of care, and outcome.

## Author contributions

KA and SI contributed to the design, writing and editing the paper. Both authors approved the submitted version.

## Conflict of interest

The authors declare that the research was conducted in the absence of any commercial or financial relationships that could be construed as a potential conflict of interest.

## Publisher's note

All claims expressed in this article are solely those of the authors and do not necessarily represent those of their affiliated organizations, or those of the publisher, the editors and the reviewers. Any product that may be evaluated in this article, or claim that may be made by its manufacturer, is not guaranteed or endorsed by the publisher.
